# Mefloquine causes selective mast cell apoptosis in cutaneous mastocytosis lesions by a secretory granule‐mediated pathway

**DOI:** 10.1111/exd.14651

**Published:** 2022-08-04

**Authors:** Maria Lampinen, Eva Hagforsen, Simone Weström, Anna Bergström, Kerstin Hamberg Levedahl, Aida Paivandy, Paola Lara‐Valencia, Gunnar Pejler, Ola Rollman

**Affiliations:** ^1^ Department of Medical Sciences, Dermatology and Venereology Uppsala University Uppsala Sweden; ^2^ Department of Medical Biochemistry and Microbiology Uppsala University Uppsala Sweden; ^3^ Department of Dermatology Uppsala University Hospital Uppsala Sweden; ^4^ Department of Public Health and Caring Sciences Uppsala University Uppsala Sweden; ^5^ Department of Immunology, Genetics and Pathology Uppsala University Uppsala Sweden; ^6^ Present address: Department of Immunology, Genetics and Pathology Uppsala University Uppsala Sweden

**Keywords:** apoptosis, mast cells, mastocytosis, mefloquine, secretory granules

## Abstract

Mastocytosis is a KIT‐related myeloproliferative disease characterised by abnormal expansion of neoplastic mast cells (MC) in the skin or virtually any other organ system. The cutaneous form of adult‐onset mastocytosis is almost invariably combined with indolent systemic involvement for which curative therapy is yet not available. Here we evaluated a concept of depleting cutaneous MCs in mastocytosis lesions ex vivo by targeting their secretory granules. Skin biopsies from mastocytosis patients were incubated with or without mefloquine, an antimalarial drug known to penetrate into acidic organelles such as MC secretory granules. Mefloquine reduced the number of dermal MCs without affecting keratinocyte proliferation or epidermal gross morphology at drug concentrations up to 40 μM. Flow cytometric analysis of purified dermal MCs showed that mefloquine‐induced cell death was mainly due to apoptosis and accompanied by caspase‐3 activation. However, caspase inhibition provided only partial protection against mefloquine‐induced cell death, indicating predominantly caspase‐independent apoptosis. Further assessments revealed that mefloquine caused an elevation of granule pH and a corresponding decrease in cytosolic pH, suggesting drug‐induced granule permeabilisation. Extensive damage to the MC secretory granules was confirmed by transmission electron microscopy analysis. Further, blockade of granule acidification or serine protease activity prior to mefloquine treatment protected MCs from apoptosis, indicating that granule acidity and granule‐localised serine proteases play major roles in the execution of mefloquine‐induced cell death. Altogether, these findings reveal that mefloquine induces selective apoptosis of MCs by targeting their secretory granules and suggest that the drug may potentially extend its range of medical applications.

## 1 INTRODUCTION

Mastocytosis is a heterogenous group of rare, but possibly underdiagnosed, diseases characterised by clonal expansion of mast cells (MCs) in various body organs.^1^, ^2^ The accumulation of atypical MCs in mastocytosis is almost exclusively caused by point mutations in *KIT*, which encodes the transmembrane tyrosine kinase protein (CD117) that binds stem cell factor, a major growth factor for MCs.^3^ The most common mutation in mastocytosis is a D816V substitution in the kinase domain of KIT, but other activating mutations may also lead to constitutive phosphorylation of the receptor and subsequent hyperproliferation of MCs.^3^

Like their normal counterparts, MCs in mastocytosis are multifaceted and capable of secreting a wide panel of potent mediators. These molecules are either preformed and released from secretory granules (e.g. histamine, proteases, lysosomal enzymes, cytokines) or newly synthesised as a result of MC activation (e.g. prostaglandins, leukotrienes, cytokines, chemokines, and growth factors).^4^ Hence, infiltration of neoplastic MCs in mastocytosis patients may not only cause organ damage but also systemic illness due to harmful release of a broad range of pro‐inflammatory and other bioactive substances. A potential strategy to ameliorate symptoms associated with mastocytosis may therefore be to eliminate excessive MC populations without injuring other cell types.^5^, ^6^

The skin is the most commonly involved organ in mastocytosis. Overall, at least 80% of all mastocytosis patients have cutaneous manifestations which vary with the age of onset.^7^ Children typically present with solitary skin tumors (mastocytoma) that resolve with time, whereas most adult patients are affected by persistent and extensive, itchy skin lesions that may urticate upon rubbing. The adult‐onset variant, formerly called urticaria pigmentosa but now entitled maculopapular cutaneous mastocytosis (MPCM), is usually associated with indolent systemic mastocytosis. Histopathologically, MPCM is characterised by infiltration of round or spindle‐shaped MCs in the upper (papillary) layer of the dermis close to adnexal structures, nerve fibres and capillaries.^8^ The cells are morphologically abnormal and typically express aberrant immunophenotypic markers such as CD2 and CD25.

Further to skin discomfort and extra‐cutaneous organ manifestations, MPCM patients with associated systemic mastocytosis may suffer from general mediator‐induced symptoms such as heat intolerance, flushing, hypotension, nausea, anaphylaxis and fatigue.^9^ There is also a potential risk of transformation to a more aggressive phenotype of mastocytosis or hematologic malignancy. However, the vast majority of patients with adult‐onset MPCM maintain a stable and chronic course.^7^

Symptomatic therapies for cutaneous mastocytosis include topical corticosteroids and oral H1‐antihistamines. Phototherapy may also ease the skin symptoms although concerns have been raised that UV treatment may further increase the risk of skin melanoma in mastocytosis patients.^10^ Cytoreductive drugs including imatinib, midostaurin and avapritinib are occasionally used in selected cases of severe mastocytosis.^11^ Although new generations of KIT‐targeting kinase inhibitors may pave the way for MC‐directed approaches in advanced mastocytosis, no curative therapy has yet emerged for any category of mastocytosis.^12^

In the present study, we have evaluated a MC‐depleting strategy based on the notion that (a) MCs have a higher abundance of secretory granules than most other cell types in the body, and that (b) the secretory granules contain potentially cytotoxic substances if released into the cytosolic compartment.^4^, ^5^ With the purpose of permeabilising MC granules, we have selected mefloquine, an approved drug for prophylaxis and treatment of malaria. Mefloquine is a quinoline‐based compound that interferes with lipid binding proteins, prevents heme polymerisation and inhibits protein synthesis by targeting cytosolic 80S ribosome of *Plasmodium malariae*.^13^ Furthermore, previous studies suggest that the drug exerts anti‐malarial activity by penetrating into acidic endosomal compartments of the parasite.^14^ In human cells it is known that mefloquine can diffuse over membranes after interaction with negatively charged phospholipids and also to interact with several types of receptors involved in the membrane transport of various small molecules.^13^

Since MC secretory granules are acidic, endolysosomal‐like organelles, we have reasoned that mefloquine may also have cytotoxic effects on MCs via a mechanism analogous to that on *Plasmodium malariae*. Our idea is supported by previous reports showing that mouse MCs are highly sensitive to mefloquine.^15^, ^16^ Here we show that mefloquine induces cell death of tissue‐bound MC infiltrates in mastocytosis skin biopsies ex vivo, and that the drug targets secretory granules in normal human skin MCs in vitro.

## 2 MATERIALS AND METHODS

### 2.1 Patients

In total, 22 patients (14 females, eight males) aged 20–70 (mean 49) years were recruited from Centre of Excellence in Mastocytosis (a node of the European Competence Network on Mastocytosis) at Uppsala University Hospital. The patients were classified according to the 2016 WHO criteria^17^ as having mastocytosis restricted to the skin (*n*: 5) or combined with indolent systemic mastocytosis (*n*: 14), intestinal mastocytosis (*n*: 2) or aggressive systemic mastocytosis (*n*: 1). All patients presented with widespread, reddish‐brown monomorphic skin lesions typical of MPCM, and *KIT* D816V mutation was detected in most of them (*n*: 20). The time from onset of skin symptoms was 2–47 (mean 19) years. Ongoing oral therapies included H1 (*n*: 18) and/or H2 (*n*: 5) antihistamines, either alone or combined with montelukast (*n*: 5) or sodium cromoglycate (*n*: 3).

### 2.2 Reagent

A stock solution of 50 mM mefloquine (Sigma‐Aldrich) in dimethyl sulfoxide was diluted in phosphate‐buffered saline prior to experimentation.

### 2.3 Incubation of skin biopsies with mefloquine

From each patient, two or three skin punch biopsies (3 mm in diameter) were obtained under local anaesthesia from adjacent, clinically similar mastocytosis lesions on the trunk or thighs. The biopsies were incubated with freshly prepared mefloquine at 20 μM (*n*: 11 biopsies) and/or 40 μM (*n*: 8) and/or 100 μM (*n*: 8) drug concentration in Dulbecco's modified Eagle's medium supplemented with 5% fetal calf serum (FCS) + 1% penicillin/streptomycin and L‐glutamine (all from Life Technologies). One biopsy from each patient was incubated under the same culture conditions except that mefloquine was excluded (control). The biopsies were placed in separate wells of a 48‐well plate containing 600 μl medium/well and maintained under submerged conditions in a 37°C incubator with a humidified atmosphere of 5% CO_2_ in air. Every 24 h, the culture medium was replaced with fresh medium containing the same ingredients. After 72 h, the biopsies were fixed for 24 h in 4% buffered formalin before dehydration and embedment in paraffin.

### 2.4 Immunohistochemistry and histopathology

Biopsies were cross‐sectioned (5 μm thickness) and then deparaffinised, hydrated and boiled in a pressure cooker in Reveal Decloaker (Biocare Medical). Background sniper (Biocare Medical) was used to block non‐specific background staining. For tryptase staining, sections were incubated at room temperature for 2 h with a mouse monoclonal tryptase antibody (MAB1222, Chemicon Inc.) at 1/2000 dilution. Mouse AP polymer detection kit and Vulcan Fast Red Chromogen kit 2 (both from Biocare Medical) were used for visualisation. Sections were counterstained with Mayer's haematoxylin (Histolab). Incubation with mouse IgG was used as negative control. For Ki‐67 staining, endogenous peroxidase was blocked with Peroxidazed 1 (Biocare Medical) before incubation with the primary antibody: mouse anti‐human Ki‐67 clone MIB1 (Dako, A/S). As a secondary antibody, horse anti‐mouse biotinylated IgG (Vector Labs) was used. Vectastain ABC‐Elite kit and DAB kit (both from Vector Labs) were used for visualisation. Counterstaining was performed as above. Incubation with mouse IgG was used as negative control. Standard haematoxylin and eosin (H&E) staining was performed for histopathological evaluation.

### 2.5 Evaluation of tissue staining

Sections were evaluated under a Zeiss Axiophot Imager‐Z1 microscope (immunostainings) or a Zeiss Axio Scan.Z1 slide scanner (H&E stainings) with Hitachi HV‐F203 camera and Zen Blue software (Carl Zeiss Microscopy GmbH). The number of tryptase‐positive cells in papillary dermis was counted at 400x total magnification in three sections from each biopsy. Images covering the total area of papillary dermis were taken in order to calculate the density of MCs. The number of Ki‐67‐positive epidermal cells was counted in three sections from each biopsy at 200x total magnification. The length of the dermal‐epidermal junction in each section was measured and the mean number of Ki‐67‐positive cells/mm basement membrane was calculated. Negative controls (mouse IgG antibody) did not produce any staining (data not shown).

### 2.6 Preparation of human skin mast cells

Human skin MCs were isolated as described previously with some modifications.^18^ Eyelid skin was obtained from cosmetic surgeries with informed consent of the patients. Eyelid samples from at least two individuals were combined and used in each experiment. The skin was cut into strips and treated with dispase (Roche Diagnostics Scandinavia AB) at 0.5 mg/ml, 4°C overnight. After detachment of the epidermis, the dermis was chopped into small pieces and digested with collagenase type 2 at 1.5 mg/ml, hyaluronidase type‐1S at 0.75 mg/ml (both from Sigma‐Aldrich) and DNAse at 10 μg/ml (VWR) for 1.5 h at 37°C under continuous shaking. Isolated cells were separated from the remaining tissue by three steps of filtration (pore sizes 250, 100 and 30 μm). MCs were further purified from cell suspensions by positive selection using anti‐human c‐Kit microbeads (Miltenyi Biotec) and a magnetic cell sorter separation device. MC purity in the preparations typically exceeded 95% as assessed by toluidine blue staining.

### 2.7 Incubation of human skin mast cells with mefloquine and inhibitors

Purified human skin MCs were seeded in a 96‐well plate in RPMI (Gibco/Thermo Fisher Scientific) supplemented with 10% heat‐inactivated FCS, 4 mM L‐glutamine, and antibiotics. Mefloquine (20 μM) was added to selected wells, and the plate was incubated for 2 h at 37°C in a humidified atmosphere of 5% CO_2_ in air. The effect of mefloquine on MC apoptosis was evaluated by flow cytometry analysis (below). To delineate the mechanisms behind the mefloquine‐induced apoptosis, MCs were pretreated with the pan‐caspase inhibitor Z‐VAD‐FMK (AH Diagnostics), the serine protease inhibitor Pefabloc or the tryptase inhibitor nafamostat mesylate (Sigma‐Aldrich) before stimulation with mefloquine. The ability of mefloquine to activate caspases was tested by staining of mefloquine‐treated MCs with Caspase 3/7 Green Detection Reagent (Invitrogen) and evaluation of the induced FITC fluorescence by flow cytometry. To assess whether the granule acidity has an impact on the responsiveness of MCs to mefloquine‐induced cell death, cells were pretreated with the V‐ATPase inhibitor bafilomycin A1 (Sigma‐Aldrich) at 10 nM before stimulation with mefloquine.

### 2.8 Flow cytometric analysis of cell viability

The treated MCs were incubated with annexin V and Draq7, and cell viability was evaluated by flow cytometry. MCs were gated by their forward‐ and side scatter properties, and in some of the experiments, cell purity was verified by the cell surface marker CD117 PE (Figure S1). Viable cells were identified as annexin V^−^Draq7^−^, apoptotic cells as annexin V^+^Draq7^−^, and necrotic/late apoptotic cells as annexin V^+^Draq7^+^. The cells were analysed with a two‐laser Accuri instrument (BD Biosciences). Data analysis was performed using the Kaluza 2.1 software (Beckman Coulter).

### 2.9 Measurement of granule and cytosolic pH

Changes in granule acidity were evaluated using LysoSensor Green (Invitrogen), a pH probe that accumulates in acidic organelles and whose fluorescence is dependent on protonation. Cytosolic pH was measured using the cell‐permeable fluorescent indicator BCFL‐AM (Sigma‐Aldrich). This probe produces strong fluorescence at high pH. Purified human skin MCs were treated with mefloquine (20 μM) or bafilomycin A1 (10nM) before incubation with either probe, and the fluorescence intensity in the FITC channel was measured by flow cytometry.

### 2.10 Transmission electron microscopy (TEM)

Samples were fixed in 2.5% glutaraldehyde (Ted Pella) + 1% paraformaldehyde (Merck) in PIPES pH 7.4 and stored at 4°C until further processed. Samples were rinsed with 0.1 M PBS for 10 min prior to 1 h incubation in 1% osmium tetroxide (TAAB; Berks, UK) in 0.1 M PBS. After rinsing in 0.1 M PBS, samples were dehydrated using increasing concentrations of ethanol (50%, 70%, 95% and 99.9%) for 10 min each step, followed by 5 min incubation in propylene oxide (TAAB). The samples were then placed in a mixture of Epon Resin (Ted Pella) and propylene oxide (1:1) for 1 h, followed by 100% resin and left overnight. Subsequently, samples were embedded in capsules in newly prepared Epon resin and left for 1–2 h and then polymerised at 60°C for 48 h. Ultrathin sections (60–70 nm) were cut in an EM UC7 Ultramicrotome (Leica) and placed on a grid. The sections were subsequently contrasted with 5% uranyl acetate and Reynold's lead citrate and visualised with Tecnai™ G2 Spirit BioTwin transmission electron microscope (Thermo Fisher/FEI) at 80 kV with an ORIUS SC200 CCD camera and Gatan Digital Micrograph software (both from Gatan Inc.).

### 2.11 Statistical analysis and graphs

Data were analysed using the GraphPad Prism 9.3 statistics and graphing software (GraphPad Software). For comparison of mefloquine‐treated vs. control biopsies, Wilcoxon matched‐pairs signed rank test was used. Cell‐based experiments involving three or more groups were analysed by one‐way ANOVA with post hoc Tukey's test. Statistical significance was set at *p* < 0.05.

### 2.12 Ethics statement

The study was approved by the regional ethical review board (reg. no. 2012/351). Written informed consent was provided by all patients and healthy donors before study initiation.

## 3 RESULTS

### 3.1 Mefloquine causes MC depletion in skin biopsies from mastocytosis patients

**FIGURE 1:**
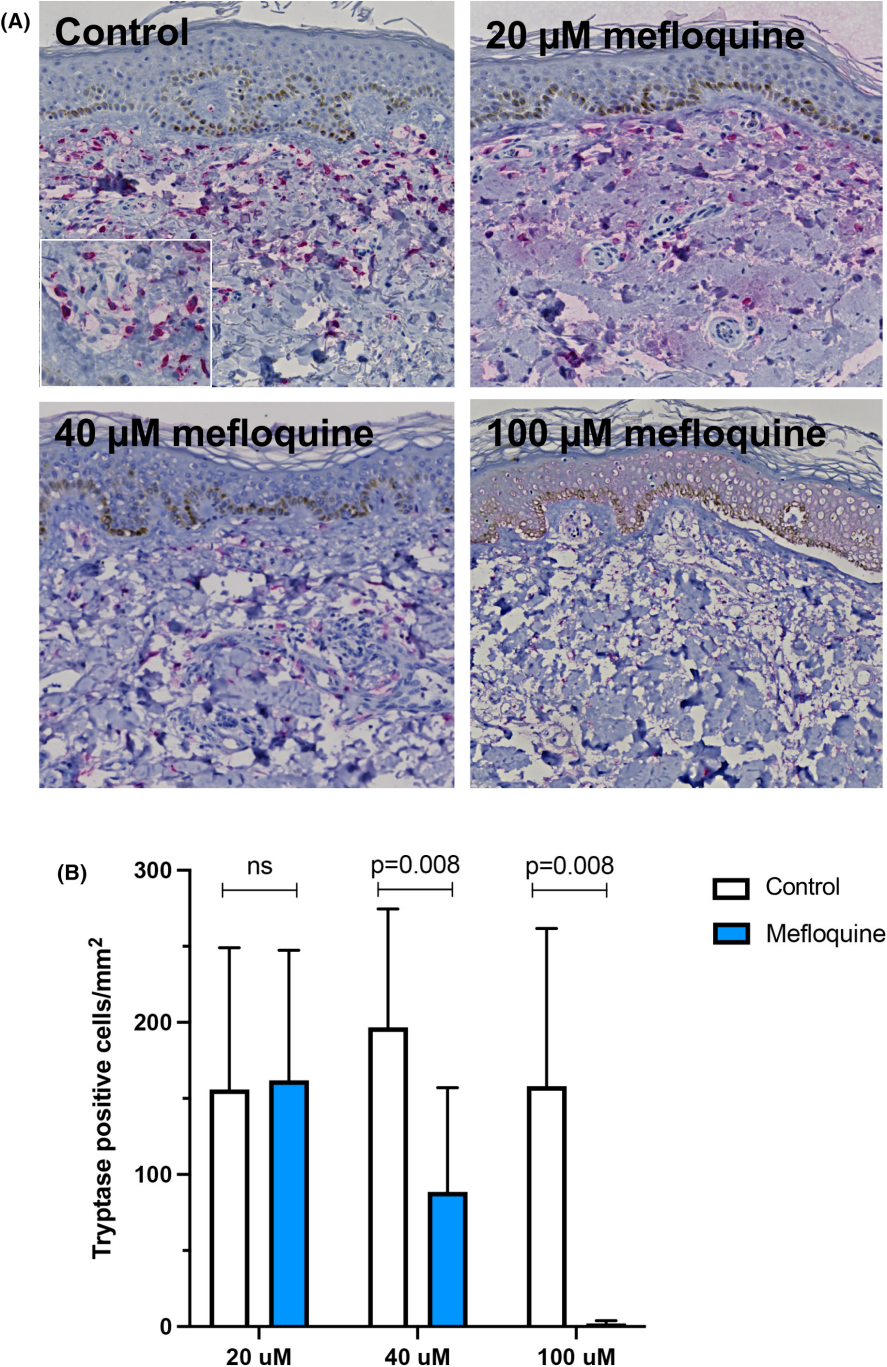
Mefloquine causes mast cell depletion in skin biopsies from mastocytosis patients. Biopsies of mastocytosis lesions were incubated in medium without (control, *n*: 27) or with mefloquine at 20, 40 or 100 μM (total *n*: 27) level for 72 h. Tissue sections were evaluated for the presence of MCs by immunostaining. (A) Representative images show a high number of tryptase‐positive dermal cells in control biopsies (insert: round and spindle‐shaped MCs), and in biopsies treated with 20 μM of mefloquine. Higher drug levels (40 and 100 μM) were associated with reduced density and almost complete depletion of positively stained cells, respectively. (B) Quantification of MCs in paired biopsy samples (total *n*: 27) treated with mefloquine vs. controls. The bars represent means + *SD* values of immunopositive cells per mm^2^ papillary dermis. Significance was tested with Wilcoxon matched pairs signed rank test.

Mastocytosis skin biopsies were maintained in culture for 72 h in the absence or presence of mefloquine at various drug concentrations. The biopsies were then processed for immunohistochemical analysis using an anti‐tryptase antibody. As shown in Figure 1A, dermal MCs were abundant in untreated skin biopsies with no apparent reduction of MC density following 20 μM drug exposure. Biopsies treated with mefloquine at 40 μM, however, showed a significant decrease in skin MC numbers (Figure 1B). Furthermore, virtually no tryptase‐stained cells were identified in biopsies exposed to 100 μM mefloquine (Figure 1A,B).

### 3.2 Effect of mefloquine on epidermal gross morphology and proliferation in mastocytosis skin biopsies

**FIGURE 2:**
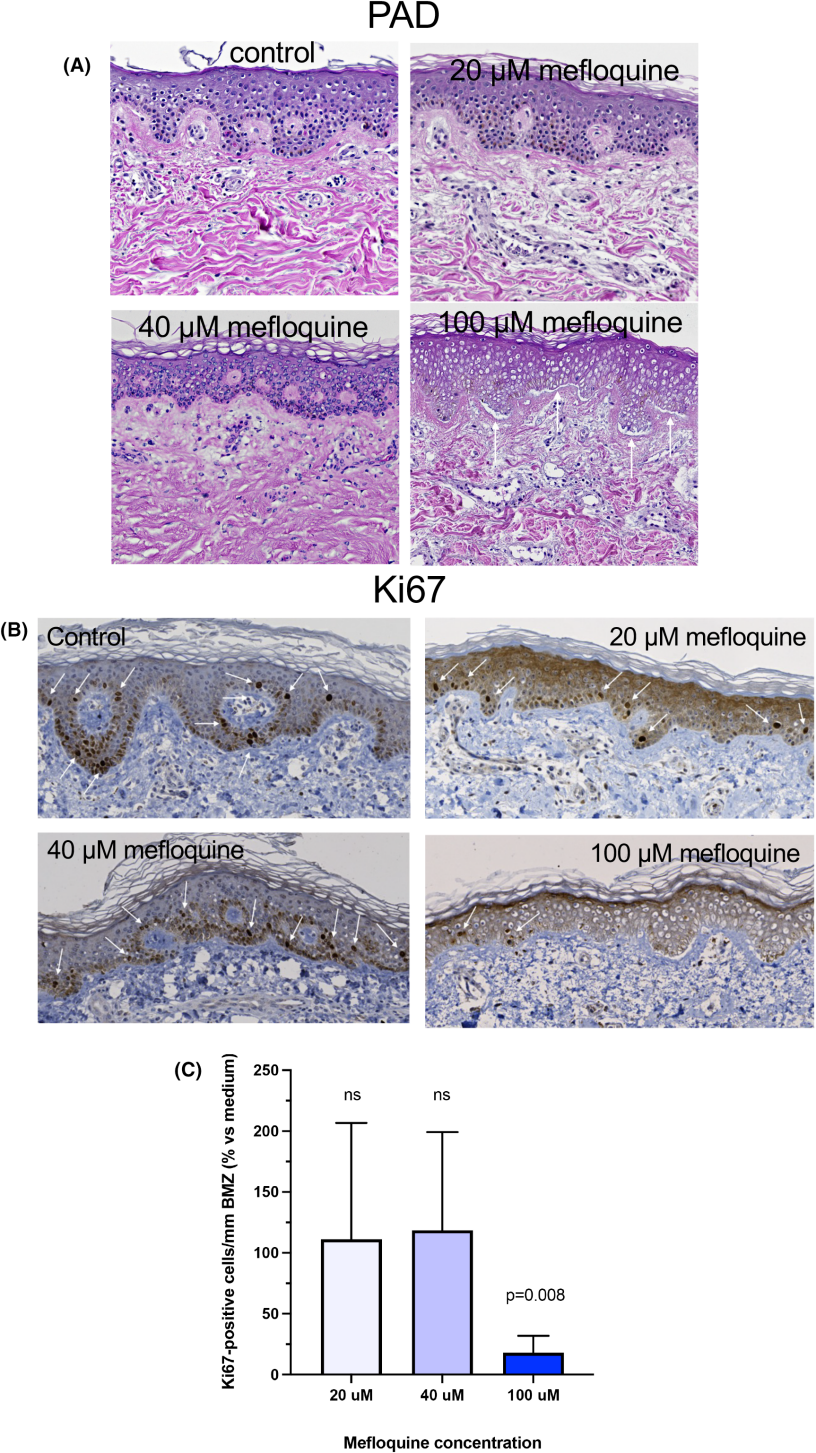
Effect of mefloquine on morphology and keratinocyte proliferation in skin biopsies from mastocytosis patients. Biopsies of mastocytosis lesions were incubated in medium without (control) or with mefloquine at 20, 40 or 100 μM concentration for 72 h. Tissue sections were evaluated following haematoxylin–eosin and Ki67 immunostaining. (A) Histopathological analysis of biopsies treated with 20 or 40 μM (*n*: 10) mefloquine showed morphological alterations comparable with control biopsies (*n*: 9). Biopsies exposed to 100 μM mefloquine displayed more extensive basal vacuolar degeneration, subepidermal cleavage (arrows) and signs of epidermal necrosis. (B) Immunohistochemical staining with anti‐Ki67 antibody demonstrated proliferative epidermal cells (arrows) in biopsies treated with mefloquine at different concentrations. (C) Ki67‐positive cell counts (means + *SD*) per mm basement membrane zone (BMZ) were not affected by mefloquine at 20 or 40 μM (*n*: 19) concentration, but markedly reduced in biopsies exposed at the 100 μM (*n*: 8) drug level. Significance was tested with Wilcoxon matched pairs signed rank test.

To assess the effect of mefloquine on skin morphology, H&E‐stained tissues were evaluated. Histopathological analysis of biopsies incubated for 72 h with mefloquine at 20 or 40 μM drug concentration showed similar epidermal features, i.e. multilayered epithelium with ordinary thickness, orthokeratosis and scattered necrotic keratinocytes (Figure 2A). Also, some degree of epidermal spongiosis was seen although generally more evident in biopsies exposed to 40 μM of mefloquine. Occasional samples also displayed subepidermal cleavage and focal degeneration of the basal layer. Overall, the morphological aberrations in epidermis were similar to those observed in control biopsies unexposed to the drug, which probably reflects the fact that the experimental conditions were not favouring keratinocytes. Clearly, the highest mefloquine concentration (100 μM) was associated with subepidermal blistering and necrosis of keratinocytes suggesting that drug toxicity added to the negative effect of suboptimal culture conditions for epidermal cells. Overall, no morphological changes were observed in the dermal compartment with the exception of the drug effects on MCs (see above).

Next, we evaluated whether mefloquine affects the expression of proliferative keratinocytes in lesional skin from mastocytosis patients. As seen in Figure 2B,C, Ki67‐positive cells were abundant and, on average, at the same level in control biopsies vs. biopsies treated with mefloquine at concentrations up to 40 μM. In contrast, a marked reduction of Ki67‐positive cells was observed following 100 μM drug exposures. The decrease in keratinocyte proliferation observed at this high drug level is consistent with the corresponding changes in epidermal morphology (see above).

### 3.3 Mefloquine causes apoptotic cell death in purified human skin MCs

**FIGURE 3:**
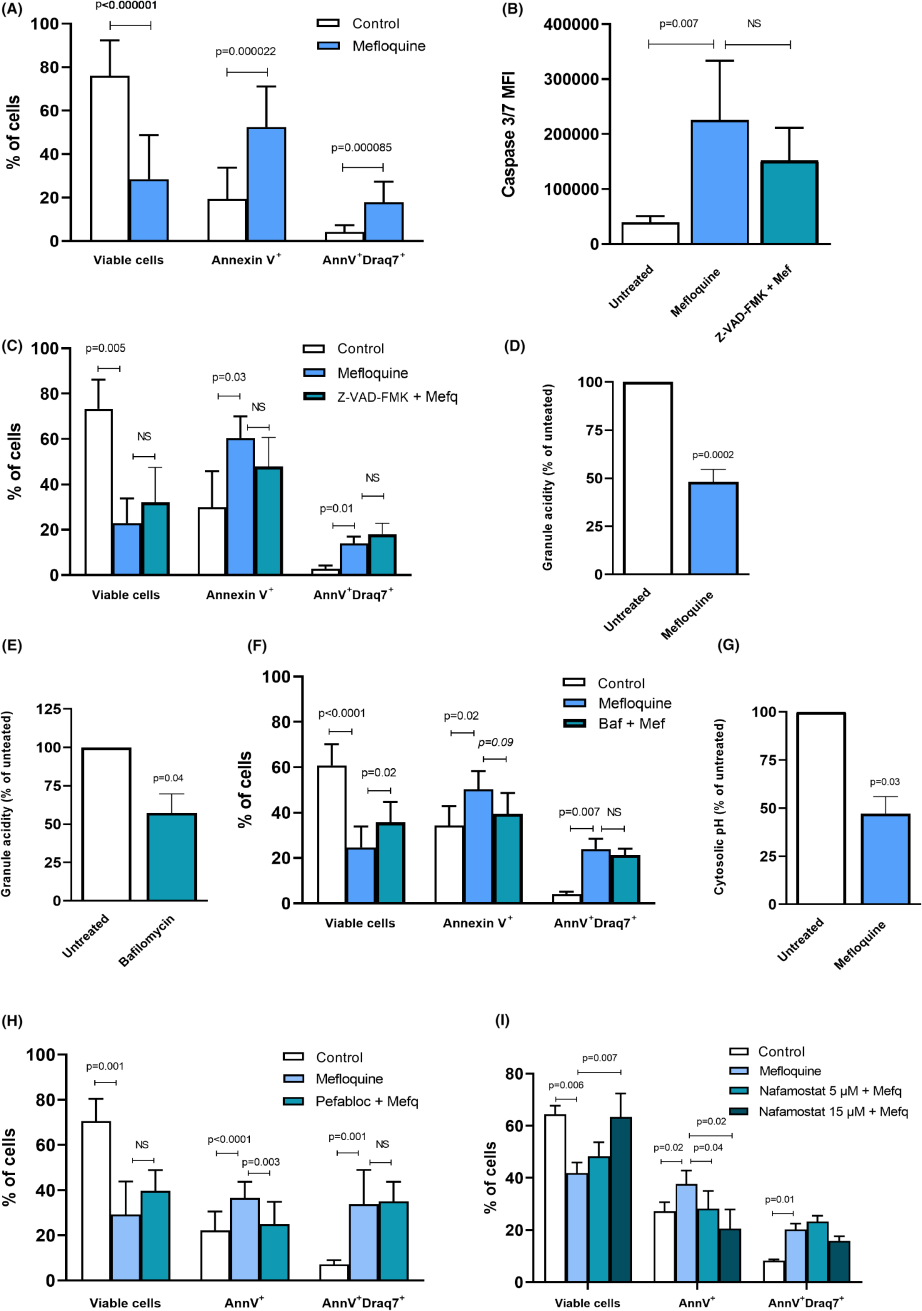
Mefloquine induces granule deacidification and serine protease‐dependent cell death in human skin mast cells. (A) Human skin mast cells treated for 2 h with mefloquine (20 μM). The effect on cell viability was assessed by staining with annexin V and Draq7. Viable cells: Ann V^−^Draq7^−^; apoptotic cells: Ann V^+^ Draq7^−^; necrotic/late apoptotic cells: AnnV^+^Draq7^+^ (*n* = 12). (B) The effect of mefloquine on caspase 3/7 activation in human skin mast cells: 1 h pretreatment with caspase inhibitor Z‐VAD‐FMK (20 μM), followed by 2 h incubation with mefloquine (20 μM). Caspase 3/7‐activation is measured as mean fluorescence intensity (MFI) of the caspase 3/7 green detection agent (*n* = 6). (C) The involvement of caspase 3/7 activation in mefloquine‐induced mast cell apoptosis was evaluated by 1 h pretreatment with caspase inhibitor Z‐VAD‐FMK (20 μM), followed by 2 h incubation with mefloquine (20 μM) and subsequent staining with annexin V and Draq7 (*n* = 6). (D) Granule acidity of human skin mast cells incubated with mefloquine (20 μM) assessed as signal intensity of LysoSensor Green (*n* = 7). (E) Granule acidity of human skin mast cells incubated with bafilomycin (10 nM) assessed as signal intensity of LysoSensor Green (*n* = 4). (F) Pretreatment with bafilomycin A1 (Baf; 10 nM) followed by 2 h incubation with mefloquine (Mef; 20 μM) and subsequent staining with annexin V and Draq7 (*n* = 5). (G) The effect on cytosolic pH of human skin mast cells by mefloquine (20 μM), assessed as signal intensity of BCFL‐AM (*n* = 3). (H) Mast cells were pretreated for 1 h with the serine protease inhibitor Pefabloc (100 μM), followed by 2 h incubation with mefloquine (20 μM) and subsequent staining with annexin V and Draq7 (*n* = 3). (I) MCs were pretreated for 1 h with tryptase inhibitor (nafamostat; 5 or 15 μM), followed by 2 h incubation with mefloquine (20 μM) and subsequent staining with annexin V and Draq7 (*n* = 3). The bar charts show mean values + *SD*. Significance was tested with Student's *t*‐test (two groups) or one‐way ANOVA with post hoc Tukey's test (three or more groups).

To approach the mechanism by which mefloquine causes a reduction in skin MC populations in situ, we next purified primary human MCs from normal skin and subjected these to mefloquine. As shown in Figure S1, the purified skin MCs were positive for KIT and produced strong granular staining with toluidine blue, hence indicating that the recovered cells represent MCs of high purity and maturity. To address the mechanism of cell death in response to mefloquine, drug‐exposed skin MCs were stained with annexin V and Draq7 to assess the proportion of apoptotic (annexin V^+^Draq7^−^) and necrotic/late apoptotic (annexin V^+^Draq7^+^) cells. As depicted in Figure 3A, mefloquine treatment reduced the proportion of viable cells, and this was accompanied by the appearance of both annexin V^+^Draq7^−^ (apoptotic) and annexin V^+^Draq7^+^ (necrotic/late apoptotic) cells. However, a larger fraction of annexin V^+^Draq7^−^ vs. annexin V^+^Draq7^+^ cells was seen, indicating that mefloquine preferentially induces apoptotic cell death of skin MCs (Figure 3A). Also, mefloquine treatment of isolated MCs resulted in activation of caspase‐3/7 as assessed by flow cytometry (Figure 3B), and the caspase activity was reduced in presence of a pan‐caspase inhibitor (Figure 3B). However, caspase inhibition did not rescue the MCs from mefloquine‐induced cell death (Figure 3C), indicating that cell death in response to mefloquine is mainly caspase‐independent.

### 3.4 Mefloquine causes an elevation of granule pH and acidification of the cytosol

Since mefloquine is known to target acidic endolysosomal compartments, both in *Plasmodium* ^19^ and in murine MCs,^15^, ^16^, ^20^ we next asked whether mefloquine could affect the endolysosome‐like secretory granules of human skin MCs. To approach this, we stained the MCs with a probe (LysoSensor) that targets lysosome‐like organelles and produces high fluorescence under acidic conditions, whereas a reduction of acidity results in loss of fluorescence. As expected, untreated MCs stained strongly with the probe, suggesting a high content of acidic secretory granules (Figure 3D). However, mefloquine treatment of the cells resulted in a profound reduction of fluorescence, indicating that mefloquine causes secretory granule deacidification (Figure 3D).

Previous studies have indicated that granule acidity in MCs is dependent on the action of V‐ATPase, as shown by loss of granule acidity in mouse MCs treated with the V‐ATPase inhibitor bafilomycin A1.^21^ To assess whether V‐ATPase also maintains the granule acidity in a human MC context, skin MCs were incubated with bafilomycin A1, followed by assessment of granule pH. This analysis revealed a profound drop in granule acidification in response to bafilomycin A1 (Figure 3E), indicating that V‐ATPase has a major role in maintaining granule acidity in human primary MCs. To assess whether the granule acidity has an impact on the responsiveness of MCs to mefloquine‐induced cell death, cells were incubated with mefloquine in the absence or presence of bafilomycin A1, followed by cell viability measurements. As seen in Figure 3F, bafilomycin A1 provided partial protection of the MCs against mefloquine‐induced cytotoxicity, indicating that granule acidity contributes to the execution of cell death.

One potential scenario to reconcile these findings could be that mefloquine induces permeabilisation of the granule membranes, leading to leakage of protons from the granules to the cytosol, hence causing a pH drop in the cytosol. To evaluate this possibility, we stained non‐ and mefloquine‐treated cells with a probe for cytosolic pH (BCFL‐AM). Indeed, mefloquine caused a pronounced acidification of the cytosol (Figure 3G), supporting a flux of protons from the granules to the cytosol in response to mefloquine.

### 3.5 Serine protease activity contributes to apoptotic MC death in response to mefloquine

Since the data above indicate that caspase activity contributes minimally to the execution of mefloquine‐induced cell death in human MCs, we considered the possibility that other types of proteolytic enzymes may account for the triggering of apoptosis. MC granules are known to contain remarkably high amounts of various serine proteases, predominantly chymases and tryptases.^4^, ^22^, ^23^ Hence, granule permeabilisation will lead to the release of proteolytic enzymes into the cytosol and probably promote cell death under such circumstances. To approach this possibility, we treated dermal MCs with a general serine protease inhibitor (Pefabloc SC) prior to mefloquine exposure. As shown in Figure 3H, Pefabloc SC caused a significant reduction in cell death in response to mefloquine, and a shift from apoptotic cell death to necrosis/late apoptosis was seen. MC secretory granules contain a range of serine proteases, out of which tryptase is highly abundant and has a broad spectrum of biological activities.^4^, ^23^ Hence, we hypothesised that tryptase may account for the cytotoxic effects of mefloquine on MCs, and we assessed this by treating the MCs with the tryptase inhibitor nafamostat. Indeed, nafamostat provided almost complete protection of the MCs against mefloquine‐induced cell death (Figure 3I), indicating that tryptase is a major effector compound in the execution of cell death in response to mefloquine.

### 3.6 Mefloquine causes major morphological alterations and damage to secretory granules of human skin MCs

**FIGURE 4:**
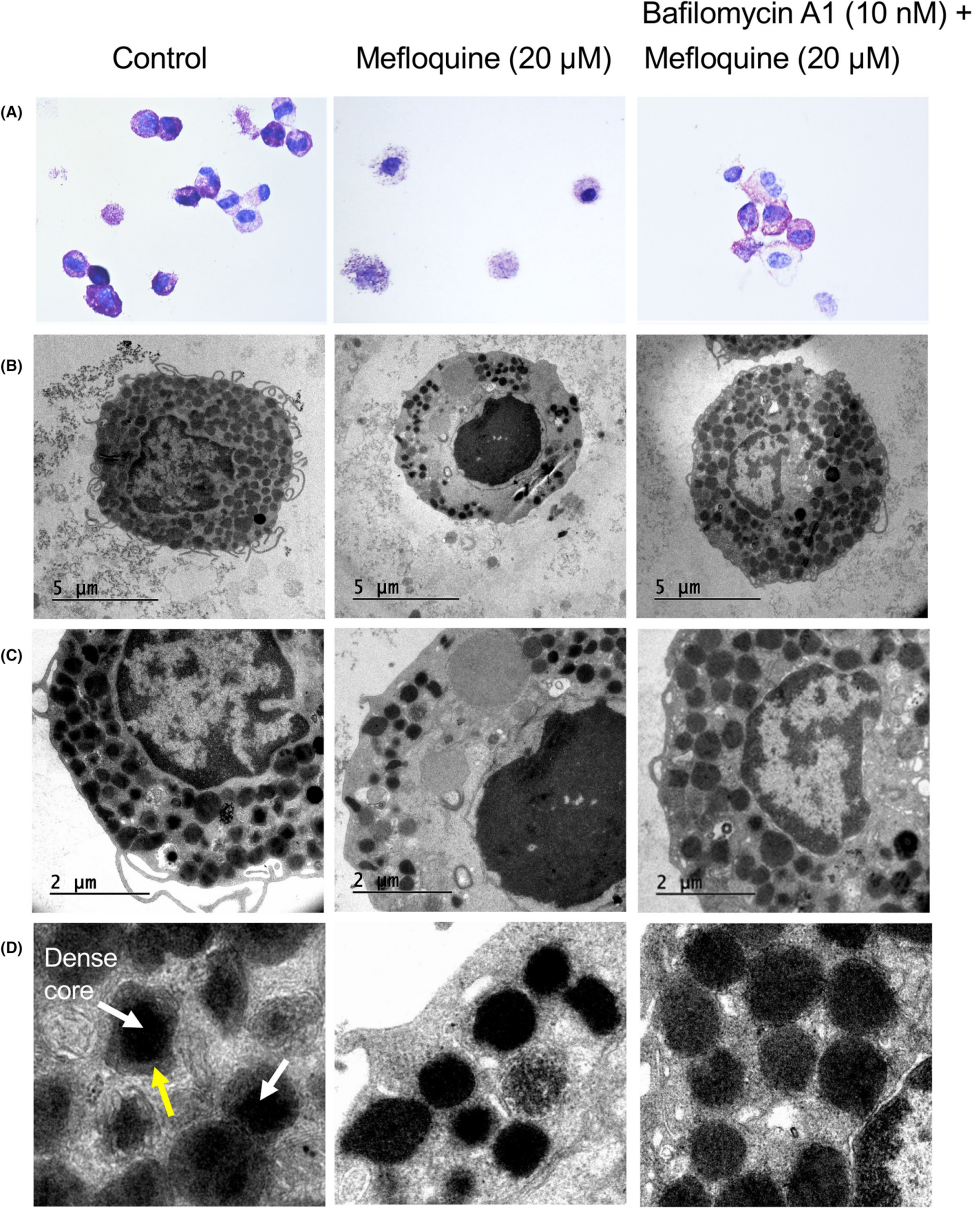
Mefloquine causes distortion of granule morphology in human skin mast cells. Human skin mast cells were incubated without or with mefloquine (20 μM), either in the absence or presence of bafilomycin A1 (10 nM) as indicated. (A) Cytospin slides were prepared and stained with toluidine blue. (B) Transmission electron microscopy analysis of mast cells treated as indicated. (C, D) Enlarged views of treated cells, highlighting the effects of mefloquine and bafilomycin A1 on mast cell granules. White arrows in left panel of (D) indicate dense core regions of granules; the yellow arrow depicts less electron dense regions in the granule periphery. Note that the separation into regions of high and low electron density is lost after treatment with mefloquine.

Light microscopy analysis (toluidine blue staining) of human dermal MCs showed that mefloquine caused major cell damage consistent with induction of cell death (Figure 4A). However, release of granules to the exterior was not apparent, suggesting that mefloquine does not induce MC degranulation. In agreement with this, only minimal release of tryptase (a granule marker) was seen in response to mefloquine (data not shown). To gain more detailed insight into how mefloquine affects MCs, we conducted transmission electron microscopy (TEM) analysis. As expected, untreated skin MCs displayed an abundance of electron dense secretory granules. Moreover, secretory granules contained central regions of particularly high electron density, i.e. representing a dense core structure (Figure 4D; white arrows), whereas the regions that are closer to the granule membranes are of less electron density (yellow arrow in Figure 4D). After treatment of MCs with mefloquine, we noted a profound nuclear condensation (a hallmark of apoptosis), thus providing further evidence that mefloquine induces apoptotic cell death in MCs (Figure 4B). Moreover, the plasma membrane was essentially intact even after mefloquine treatment, which also argues for an apoptotic mechanism of cell death. Notably, bafilomycin A1 provided protection against mefloquine‐induced effects on MC morphology and ultrastructure (Figure 4A–C), which is in line with the partial rescuing activity of bafilomycin A1 on cell viability as assessed by annexin V/Draq7 staining (Figure 3F).

We also noted that mefloquine caused extensive damage to the secretory granules (Figure 4B,C). This led to marked loss of granule density, compatible with leakage of granule constituents into the cytosol. The presence of granules with swollen appearance was also observed, indicating granule collapse (Figure 4B,C). At higher magnification, we observed that the separation of the granule contents into central regions of high electron density (dense core) and peripheral regions of lower electron density was compromised after mefloquine administration. Instead, the contents of mefloquine‐treated granules were more evenly distributed over the entire granules. Clearly, this is compatible with a mechanism where the granule contents approach the membrane lining of the granules in response to mefloquine, after which egression of granule contents to the cytosol may take place.

## 4 DISCUSSION

Mast cells play important roles in the immune system and are main effector cells in allergic diseases such as IgE‐mediated asthma and urticaria, but are also implicated in the pathophysiology of various malignancies, cardiovascular complications, type 1 diabetes, contact dermatitis and autoimmunity.^24^ In the latter disorders, activated normal MCs contribute indirectly to the pathomechanisms by secreting disease‐modifying compounds including cytokines, growth factors, proteases and lipid mediators. In contrast, mastocytosis is a distinct disease entity caused by neoplastic MCs that may infiltrate a wide range of body organs apart from releasing an abundance of bioactive mediators. Given the harmful potential of MCs in both benign and malignant MC disorders, there is a large demand for therapeutics that may reduce the negative impact of MCs in various disease states.^5^

Harmful MC activities can be suppressed through different treatment principles. In mastocytosis, current standard therapy aims to hamper the effect of individual MC mediators as exemplified by histamine‐ or leukotriene/prostaglandin receptor antagonists.^5^ However, since hyperplastic and activated MCs are capable of secreting high amounts of mediators with a broad diversity of bioactivities, an alternative—and conceptually more effective approach—might be to counteract MCs in a wider sense, e.g. by targeting cell proliferation or viability. Indeed, agents interfering with tyrosine kinases have emerged for use in severe forms of systemic mastocytosis.^25^ A disadvantage of this class of drugs, however, is that their target receptors are not MC‐exclusive but are expressed by many other cell types. Furthermore, tyrosine kinase inhibitor therapy is costly and associated with potentially severe adverse effects. An alternative strategy to reduce the number of MCs without affecting other cells would be to target a characteristic, or even better, unique feature of MCs.

Here we report a concept for targeting MCs based on their extraordinary high content of cytoplasmatic granules. These organelles share several features with lysosomes and are therefore often referred to as secretory lysosomes.^26^ For example, both granules and lysosomes are acidic, contain multiple hydrolases and have similar membrane proteins.^4^ Considering the similarities between MC granules and lysosomes, we have reasoned that lysosomotropic agents capable of permeabilising lysosome membranes^27^ may also induce leakage of granule contents into the cytosol with subsequent triggering of apoptosis as a result. Indeed, previous proof‐of‐concept studies have demonstrated that lysosomotropic agents such as Leu‐Leu‐OMe, siramesine and mefloquine exhibit preferential cytotoxicity to human MC lines and bone‐marrow‐derived MCs.^16^, ^28^, ^29^ Moreover, biopsy‐based studies by us have shown that siramesine induces apoptosis of MCs residing in normal and psoriatic skin with few negative effects on other cell types.^30^, ^31^ The present study demonstrated that a similarly acting drug, mefloquine, targets MC secretory granules and exerts a cytotoxic effect on dermal MCs in cultured mastocytosis biopsies exposed at drug levels below those affecting gross morphology.

Ex vivo skin models are technically simple and reproducible tools for in vitro studies of cutaneous biology and potential therapeutics in dermatology. In principle, two alternative culturing models are available to maintain human skin biopsies viable ex vivo: submerged or air‐exposed. The former option—as applied here—was introduced in the early 1970s and has been claimed to preserve a healthy skin appearance for nearly 2 weeks under appropriate culture conditions.^32^ More recent studies, however, have demonstrated that the normal architecture and physiology of submerged skin cultures are difficult to maintain for more than 5–9 days after incubation.^33^ The impairment of cultured skin characteristics is most obvious with regard to epidermal structure and skin barrier functions, but may be delayed if skin explants are positioned at the air–liquid interface, i.e. under more in vivo‐like conditions. Since we focused on short‐term (within 3 days) drug effects on dermal cells, we selected the submerged culture model and used a basal medium supporting maintenance of connective tissue cells rather than epidermal cells. With respect to dermal MCs specifically, a previous study by Kivinen et al has shown that the density of tryptase‐positive dermal cells diminishes over time and with a particularly deep and significant drop (by 73%) on day 14 in culture.^34^, ^35^ The authors found that the MC count was reduced by 19–21% already within 2 days irrespective of culture principle (submerged or air‐exposed). Hence, in our case it is likely that the culture procedure per se has contributed to a reduction of MC numbers in both untreated and mefloquine‐exposed biopsies.

The limited number of mastocytosis patients and their wide range of characteristics in terms of age, systemic involvement and drug exposure should be considered when interpreting our biopsy‐based results. It is likely that the large clinical heterogeneity within the patient population has contributed to the variance of data. Enrolling a larger and more homogenous mastocytosis population would have been desirable although challenging in view of such a rare and complex disease. Nevertheless, by using a paired sample design, and collecting biopsies from neighbouring and clinically inseparable lesions, we controlled for differences between patients. Another unresolved issue of our study is whether mefloquine affects all MCs similarly or if there is a difference between normal and atypical MC phenotypes in view of drug sensitivity.

Mechanistically, our data suggest that mefloquine causes granule permeabilisation in MCs. This is firstly supported by the noted elevation of granule pH in response to mefloquine, which was accompanied by a corresponding drop in cytosolic pH. Clearly, these findings are in line with leakage of protons from permeabilised granules into the cytosol. In further agreement with a granule permeabilisation mechanism, TEM analysis revealed that mefloquine‐treated granules were dramatically depleted of content, again in agreement with granule permeabilisation leading to leakage of granule contents into the cytosol. Further, our data show that tryptase has a major role in executing apoptosis downstream of mefloquine treatment. Tryptase is normally localised within the granules and is thereby inaccessible to the cytosolic pro‐apoptotic machinery. Hence, the most likely scenario behind the proapoptotic impact of tryptase following mefloquine treatment is that tryptase has leaked from the granules to the cytosol, as a consequence of mefloquine‐dependent granule permeabilisation. In addition to the marked role of tryptase in executing mefloquine‐dependent apoptosis in MCs, it is conceivable that the actual acidification of the cytosol following granule permeabilisation may have an impact on cell death. In support of this notion, inhibition of granule acidification was shown to dampen the ability of mefloquine to induce MC apoptosis. It is known that certain DNAses are activated by a decrease in cellular pH,^30^, ^31^, ^32^, ^33^ and it is thus plausible that activation of such enzymes may contribute to the cell death following mefloquine treatment of MCs. It is also notable that mefloquine‐induced cell death was not affected by caspase inhibition, i.e. contrasting to classical apoptosis.

Taken together, our study reveals that mefloquine targets secretory granules and reduces the number of infiltrating MCs in mastocytosis skin lesions ex vivo. Given the wide array of detrimental actions of MCs in mastocytosis and the limited treatment options, mefloquine or other lysosomotropic agents may have future roles to play in medicine. Further development and testing are required to determine the potential usefulness and efficacy‐to‐risk relationship of mefloquine as a repurposing candidate drug in mastocytosis.

## AUTHOR CONTRIBUTIONS

ML planned and performed experimental work, and contributed to the writing of the manuscript; EH performed experimental work, and contributed to the writing of the manuscript; SW performed experimental work; AB performed tissue samplings; KHL coordinated and facilitated patient recruitment; AP contributed to the design of the study; PLV carried out histopathological analyses; GP conceived and planned the study, provided laboratory resources and wrote the manuscript; OR conceived and planned the study, performed tissue samplings and wrote the manuscript.

## CONFLICT OF INTEREST

The authors declare no conflict of interest.

## Supporting information


**Figure S1** Flow cytometry analysis of human skin mast cells: (A) A gate was set on single cells. Mast cells were identified by (B) their forward scatter (FSC) and side scatter (SSC) properties and (C) the expression of CD117. (D) Representative photo of cytocentrifuge preparations of mast cells stained with toluidine blue. Mast cell purity typically exceeded 95%.

## Data Availability

All data are included in the manuscript.
