# Supplementary material for: Mefloquine causes selective mast cell apoptosis in cutaneous mastocytosis lesions by a secretory granule‐mediated pathway
Source: Exp Dermatol. 2022 Aug 4;31(11):1729–40. doi: 10.1111/exd.14651 (PMC9804232; doi:10.1111/exd.14651)
Supplement: Supplementary file 1 — Figure S1 Flow cytometry analysis of human skin mast cells: (A) A gate was set on single cells. Mast cells were identified by (B) their forward scatter (FSC) and side scatter (SSC) properties and (C) the expression of CD117. (D) Representative photo of cytocentrifuge preparations of mast cells stained with toluidine blue. Mast cell purity typically exceeded 95%. [file EXD-31-1729-s001.docx]

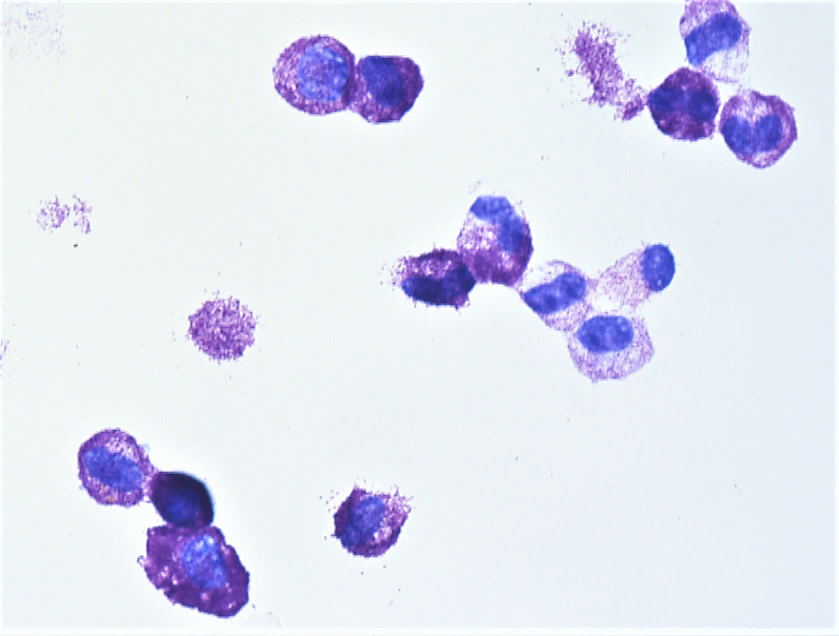


**CD117 PE-A**

**Supplementary Figure 1.** Flow cytometry analysis of human skin mast cells: A) A gate was set on single cells. Mast cells were identified by B) their forward scatter (FSC) and side scatter (SSC) properties and C) the expression of CD117. D) Representative photo of cytocentrifugation of the mast cell preparation, stained with toluidine blue. Mast cell purity typically exceeded 95%.
